# Effect of team training and monitoring on the rate of failed mid and low cavity vacuum extraction: a hospital based intervention study

**DOI:** 10.1186/s12884-019-2257-z

**Published:** 2019-03-29

**Authors:** Kristina Pettersson, Magnus Westgren, Rebecca Götze-Eriksson, Gunilla Ajne

**Affiliations:** 1 Department of Obstetrics and Gynecology, Karolinska University Hospital at Huddinge, K57, 141 86 Stockholm, Sweden; 2 Clintec, Karolinska Institute, Stockholm, Sweden

**Keywords:** Vacuum extraction, Failed extraction, Team training, Hawthorne effect, Monitoring

## Abstract

**Background:**

Clinical team training has been advocated as a means to improve delivery care, and failed extractions is a suggested variable for clinical audit in instrumental vaginal delivery.

Other activities may also have intended or unintended effects on care processes or outcomes.

**Methods:**

We retrospectively observed 1074 mid and low vacuum extraction deliveries during three time periods (prevalence periods): Baseline (period 0), implemented team training (period 1 and 2) and monitoring of traction force during vacuum extraction (period 2). Our primary outcome was failed extraction followed by emergency cesarean section or obstetric forceps delivery.

**Results:**

The prevalence proportion (relative risk) of failed extraction decreased significantly after implementation of team training, from 19% (period 0) to 8 % (period 1), corresponding to a relative risk of 0.48 [0.26–0.87]. The secondary procedural outcome *complicated delivery* (duration > 15 min *or* number of pulls > 6, *or* cup detachment > 1) was decreased in period 2 compared to period 1, RR 0.42 [0.23–0.76]. Secondary clinical (neonatal) outcome were not affected.

**Conclusion:**

Clinically based educational efforts and increased monitoring improved procedural outcome without improving neonatal outcome. The study design has inherent limitations in making causal inference.

**Electronic supplementary material:**

The online version of this article (10.1186/s12884-019-2257-z) contains supplementary material, which is available to authorized users.

## Background

Good practice guidelines for obstetric care [1–3] has become increasingly important following reports on poor judgement as a cause of perinatal mortality and severe morbidity [4–7]. Furthermore, in order to meet the possible negative consequences of increasing rates of cesarean section [8], the argument for safe operative vaginal delivery is crucial. In Sweden, a national initiative of educational and policy interventions has been introduced to generally improve delivery care [9], including vacuum extraction delivery, but evaluating these policy changes has proven to be a significant challenge, and the effects on perinatal asphyxia is ambiguous [10]. Some studies, however, have shown measurable results from educational endeavors, such as a 50% decreased risk of obstetric anal sphincter injuries in Norway [11]; a decreased prevalence of severe asphyxia following the implementation of a national educational program in Australia [12], and a simultaneous decrease in emergency cesarean section and operative vaginal delivery in Sweden [13]. Some of the components of effective team training are in-house-setting, multi-professional teams, realistic training tools, and regular recurrence [14]. Suitably designed educational programs can potentially increase quality and enhance safety, but their effects require evaluation.

One possible quality and safety indicator is failed extraction, since this has been identified as a risk factor for adverse perinatal outcome [15–19], and recommended as audible standards by Royal College of Obstetricians and Gynaecologists [1]. A further risk increase seems to occur when multiple modes are needed, that is vacuum *and* forceps attempt preceding emergency cesarean section [18, 20].

This pre-post intervention study aimed to observe measurable effects on the safety of vacuum extraction deliveries when introducing team training and increased monitoring aimed at vacuum extraction. We compared three time periods characterized by different clinic-based activities that might influence how vacuum extraction is performed: Baseline (period 0), implemented team training (period 1 and 2) and monitoring of traction force during vacuum extraction (period 2). These activities are presented in more detail in the Material and methods section. Our primary hypothesis was a decreased failed extraction rate following team training (period 1), due to an increased adherence to guidelines in the delivery team, including obstetricians´ technique. In period 2, after the additional introduction of objective traction force measurement, we wanted to observe any alteration in the failed extraction rate.

## Methods

**Fig. 1:**
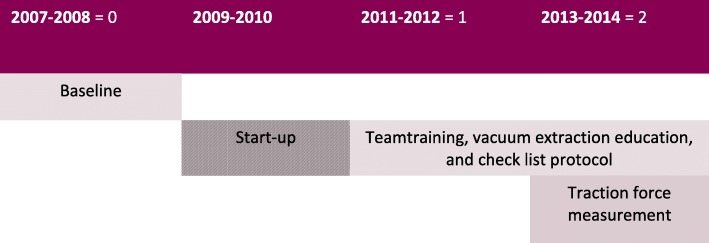
Timeline of exposure periods

We retrospectively included all women at Karolinska University hospital in Huddinge, Sweden who underwent delivery by complete or attempted vacuum extraction at fetal head station low or mid during three time periods, *N* = 1074. Period 0: 2007–2008; Period 1: 2011–2012; Period 2: 2013–2014. (2009–2010 are excluded as they were transition years when the educational program was in a start-up phase). See Fig. 1 for timeline of exposure. The design is conceptually equivalent to a cross-sectional study with three prevalence periods. We identified the cohorts retrospectively in the electronic medical records system (Obstetrix©). Each individual vacuum extraction protocol was examined to confirm and separately classify mid vs low deliveries according to American and British guidelines [1, 2], since this distinction of low vs mid is not available in Swedish registers. Vacuum extractions in Sweden are nearly exclusively carried out by doctors, and for non-outlet extractions metal cups are often used, nearly exclusively Bird metal cup size 50 mm. In this material only one non-outlet extraction was carried out by a midwife. All extractions reported as outlet extractions were excluded, since clinical experience and our previous data indicate that outlet extractions seldom lead to failed extractions or other complications [21]. Preterm delivery (< 36 full weeks of gestation) and multiple pregnancies were excluded. See Additional file 1 for flow chart. Data collection of clinical variables was carried out by two experienced medical doctors (residents) in obstetrics and gynecology. In addition, we identified all cases with emergency cesarean section at fully dilated cervix by scrutinizing the partogram and medical record text of all emergency cesarean section records. A description is presented of the overall registered rates of vacuum extraction, the proportion of non-outlet (low and mid) extractions, and emergency cesarean sections at the clinic during the time periods.

### Exposure (interventions)

Period 0 constitutes a conceptual null exposure, that is the vacuum extraction management prior to implementing active structured measures to improve delivery care. These structured improvement actions therefore form the exposure or intervention in period 1, and are described in detail in Additional file 2. In summary, they consist of a time out check list for vacuum extractions, annually recurrent clinical setting multi-professional team training based on vacuum extraction cases, and education regarding risk factors for complicated extractions. The team training sessions are led by a senior consultant and experienced midwife, and set in a delivery room using a mock pelvis and live actor from the staff, with direct feed back from a peer group rather than video recordings. The exposure or intervention of period 2 is the research based introduction of an electronical extraction handle which objectively measures and records the magnitude and duration of traction force employed in metal cup extractions.

All term, singleton deliveries were eligible, and there was no feedback on traction force to the obstetrician. These measurements infer an unintended but foreseen monitoring of individual performance. Both exposures may also be afflicted with a general highlighting effect that we believe is inevitable when all personnel are participating in a specific project, a concept known as the Hawthorne effect [22]. The equipment and results of traction force measurement have been described in detail by the authors elsewhere [21, 23].

### Clinical characteristics

Maternal variables included age (years); short stature (< 1.55 m); obesity (Body mass index ≥30) and parity (0/1). Obstetric variables included gestational age (days); use of synthetic oxytocin (y/n); epidural analgesia (y/n); duration of first and second stage (minutes); fetal head station identified in the vacuum extraction protocol (low vs mid); fetal head position (occiput anterior or non-occiput anterior); indication of the procedure (dystocia or fetal distress, where dystocia included maternal fatigue and prophylactic); large for gestational age ≥ 4500 g. A registered double diagnosis of indication was scrutinized by the data collectors, requiring either a non-reassuring cardiotocogram or an elevated scalp lactate to be indexed as fetal distress); duration of the extraction (minutes from beginning of extraction until final cup removal).

### Outcome variables

The primary outcome variable, failed extraction, was identified in the medical records. Failed vacuum was defined as an attempt at extraction followed by forceps, emergency cesarean section, or both. The *procedural* secondary outcomes were failed extraction with more than one sequential mode of delivery (followed by *both* forceps *and* cesarean section), as well as complicated extractions, defined as one or more of the following: more than 15 min duration; more than six pulls, or more than one cup detachment. Clinical secondary outcome included shoulder dystocia (y/n); asphyxia (pH < 7,0 y/n); APGAR score < 7 at 5 min (y/n); admission to neonatal intensive care unit (y/n) and anal sphincter injury (y/n).

### Statistical analyses

Clinical characteristics were analyzed using descriptive frequency measures and hypothesis tests as applicable: Mean (standard deviation) and t-test for normally distributed numerical variables, median (interquartile range) Rank sum and Kruskal-Wallis for skewed continuous data, and Chi^2^ for categorical data. As advised for analytical cross-sectional studies, Poisson regression was used for multivariate analysis of association between the exposure(s) and outcome, providing an approximation of a relative risk estimate based on prevalence proportions [24, 25]. *P*-values in the multivariate analyses were Bonferroni corrected due to multiple testing. The primary and secondary procedural outcomes were adjusted by year of birth. No other available variables were identified as confounders. We have not performed repeat measurement calculations as generalized estimating equation or mixed models effect, as the recurring individuals constituted only 1 % of the study sample. Confidence intervals regarding differences involving *total* delivery numbers in the sample (all vacuum extractions, emergency cesarean section) were calculated by hand, since they are based on group data rather than individuals.

### Missing data

There was no missing data in primary outcome. The secondary procedural outcome complicated extraction was missing in 3 %. Maternal body mass index was the only clinical variable missing in more than 1 %: (7.5%). Since these were not primary variables, we decided not to handle this missing data further.

## Results

The proportion of failed extractions among low and mid extractions was 12 %, and sequential instruments were used in 1.5%. In approximately half of the cases, the indication for vacuum extraction was dystocia. More than half of the extractions were carried out at mid cavity fetal head station.

Maternal and obstetric clinical characteristics are shown in Table 1. Epidural and oxytocin use, parity, occiput anterior position, obesity and large for gestational age showed no significant differences between the three exposure periods. All other independent variables displayed some degree of difference throughout the study periods.

**Table 1:** Clinical characteristics

	Period 0 (*n* = 328)	Period 1 (*n* = 370)	Period 2 (*n* = 376)	All *N* = 1074	
Age, years^§^	31 (5)	31 (5)	30 (5)	31 (5)	p^a^ NS *p*^b^ < 0.05 p^c^ NS
BMI ≥ 30	24 (9)	42 (12)	37 (10)	103 (10)	NS
Height cm ≤ 1,55	9 (3)	27 (7)	20 (5)	56 (6)	*p*^a^ < 0.05 p^b^ NS p^c^ NS
Nulliparous	264 (80)	297 (80)	294 (78)	855 (80)	NS
GL, days^#^	283 (276–289)	283 (276–288)	280 (273–287)	281 (275–287)	p^a^ NS *p*^b^ < 0.01 *p*^c^ < 0.01
Duration 1st stage min median^#^	480 (300–690)	540 (360–750)	480 (300–660)	510 (330–690)	*p*^a^ < 0.05 *p*^b^ < 0.01 p^c^ NS
Duration 2nd stage min median^#^	120 (60–210)	150 (60–210)	165 (90–230)	150 (60–210)	*p*^a^ < 0.05 p^b^ NS *p*^c^ < 0.01
Epidural	245 (75)	283 (77)	297 (79)	825 (77)	NS
Oxytocin	304 (93)	348 (94)	354 (94)	1006 (94)	NS
Indication dystocia	168 (51)	227 (62)	202 (54)	597 (56)	*p*^a^ < 0.01 *p*^b^ < 0.05 p^c^ NS
Fetal head station mid	190 (58)	248 (68)	231 (61)	678 (62)	*P*^a^ < 0.01 p^b^ NS p^c^ NS
Birthweight ≥ 4500 g	10 (3)	15 (4)	9 (2)	34 (3)	NS
Position non-OAP	37 (11)	52 (14)	54 (15)	143 (13)	NS
Duration > 15 min^*^	15 (5)	44 (12)	21 (6)	80 (7)	*p*^a^ < 0.01 *p*^b^ < 0.01 p^c^ NS
Nr pulls > 6^*^	34 (11)	43 (12)	23 (6)	100 (10)	p^a^ NS p^b^ < 0.01 p^c^ < 0.05
Cup detachment > 1^*^	24 (7)	15 (4)	10 (3)	49 (5)	p^a^ NS p^b^ NS p^c^ < 0.01

^§^t-test mean (sd) #Wilcoxon Rank sum and Kruskal-Wallis median (25–75). All others Chi^2^

a = period 0 vs 1, b = period 1 vs 2, c = period 0 vs 2

^*^Variables part of the composite secondary outcome variable *complicated extraction*

Primary and secondary procedural outcome is shown in Table 2. The primary outcome failed vacuum extraction decreased significantly in period 1 compared to period 0, adjusted relative risk 0.48. A significant decrease in complicated extractions was seen between period 1 and 2, adjusted relative risk 0.42.

**Table 2:** Primary and secondary procedural outcome, relative risk

	Period 0 n(%)	Period 0 vs 1[CI]	Period 1 n(%)	Period 1 vs 2[CI]	Period 2 n(%)	Period 0 vs 2[CI]
Failed extraction	61 (19)		28 (8)		37 (10)	
RR, crude		0.41 [0.27–0.62] p < 0.001		1.3 NS		0.53 [0.36–0.77] p 0.001
RR_a_		0.48 [0.26–0.87] p 0.016		NA		0.59 NS
Complicated extraction^*^	55 (17)		77 (22)		39 (11)	
RR, crude		1.3 NS		0.47 [0.33–0.68] *p* < 0.001		0.61 [0.42–0.90] p 0.012
RR_a_		NA		0.42 [0.23–0.76] p 0.004		0.53 NS

Poisson regression. P-value below 0.017 (0.05/3 for Bonferroni correction) considered significant

RR_a_: adjusted by year of birth

^*^duration > 15 min or number of pulls > 6, or cup detachment > 1

The secondary outcome multiple sequential modes of delivery could not be calculated due to few cases: thirteen cases in period 0, and two cases in period 1 and 2 respectively. Secondary clinical outcome showed no significant differences (Additional file 3: Table S1).

**Fig. 2:**
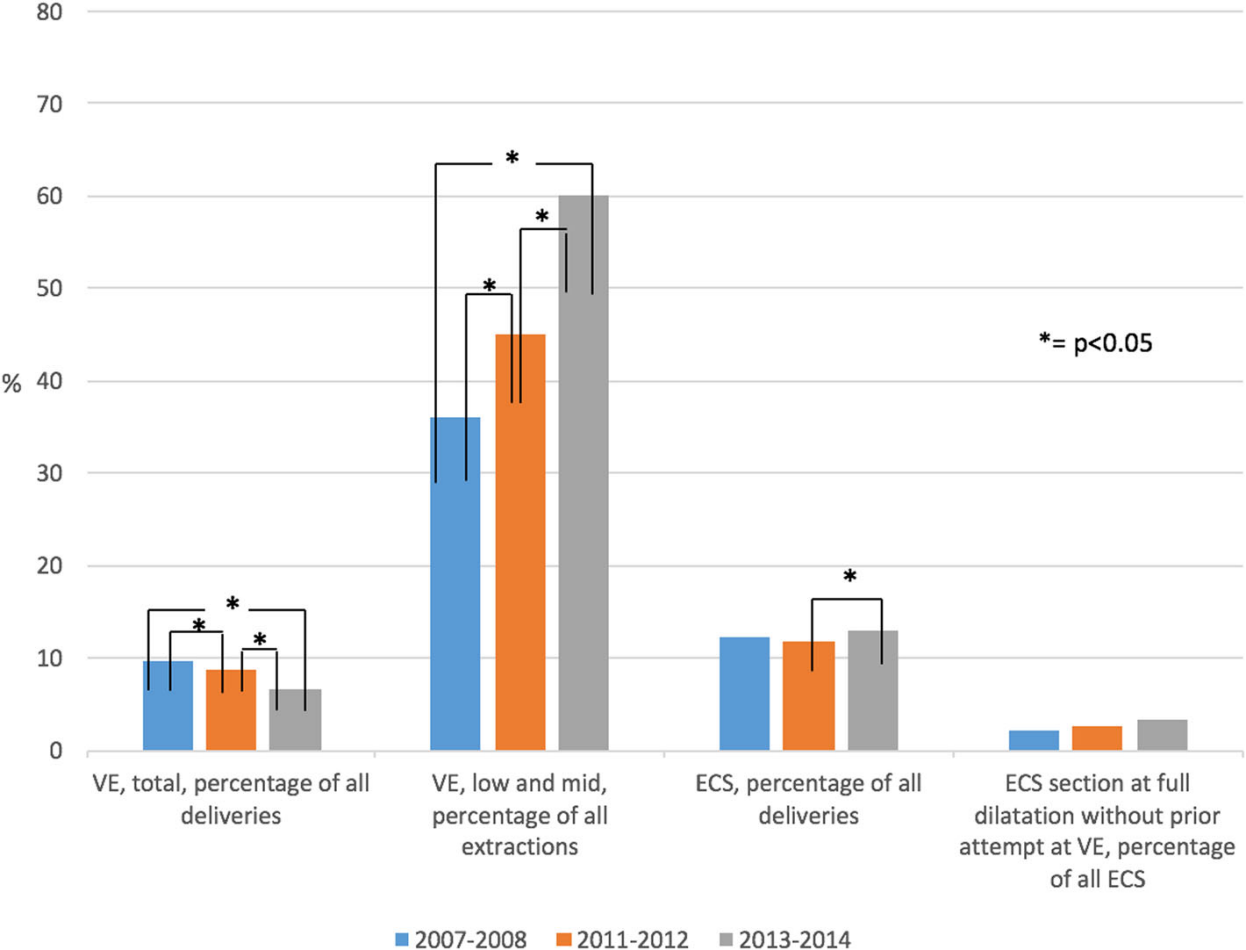
Mode of delivery. Mode of delivery (total VE, proportion low and mid VE, total ECS and proportion ECS at fully dilated cervix *without* prior attempt at VE) during the exposure periods. ^*^ = *p* < 0.05

The distribution of operative modes of delivery in the study sample is shown in Fig. 2. The total rate of vacuum extraction has decreased; period 0 9.7%; period 1 8.8%; period 2 6.5% (*p* < 0.05). The proportion of low and mid extractions (as compared to outlet) increased markedly throughout the periods: period 0 36%; period 1 45%; period 2 60% (p < 0.05). There was no significant increase in emergency cesarean sections between the first two periods, but from period 1 to period 2 the rate increased by 1.3% units (p < 0.05). The rate of emergency cesarean section *without* prior attempt of vacuum extraction at fully dilated cervix and engaged fetal head showed a non-significant increasing trend: period 0: 2.1%; period 1: 2.7%; period 2: 3.4%.

## Discussion

### Main findings

The main finding of this study is the decreased frequency of failed extractions after introduction of clinical team training, and a decreased risk of complicated extraction at the additional introduction of monitoring through traction force measurement. The total frequency of vacuum extraction also decreased during the whole study period, while the proportion of emergency cesarean section was increased.

### Interpretation (in the light of other evidence)

To the best of our knowledge, no previous study has specifically investigated the effect of clinical team training or monitoring traction force on failed extraction rates. However, previous studies have shown positive effects of team training [14], and educational efforts have been found to decrease the frequency of anal sphincter injury and asphyxia, and to increase rates of normal delivery [11–13].

The subsiding effect on the failure rate after period I complicates the interpretation; does the team training effect fade out, or is it counteracted by an opposing effect of traction force measurement? Or was there no true exposure effect between period 0 and period I, but merely a regression towards the mean and residual confounding? Interestingly, the proportion of low and mid cavity extractions increased during the study period, a situation with known increased risk for failure compared to outlet vacuum extraction. Unfortunately, the design does not allow for a separate analysis of the two exposure effects.

An increased emergency cesarean section rate as the price to pay for fewer (failed) extractions may seem intuitive, but this observation is not necessarily a direct effect of fewer vacuum extractions; emergency cesarean sections performed at a *fully dilated cervix without* a prior attempt at vacuum or forceps did not increase significantly during the studied time periods. This may indicate that obstetricians did not become more prone to choose cesarean section instead of vacuum, since the increase in emergency cesarean section rates were not predominantly in the category that could have undergone an attempted extraction. A recent Swedish hospital based study on approximately 8000 nulliparous women further support the notion that obstetric care enhancing efforts can lead to decreased vaginal instrumental delivery *without* a simultaneous increase in emergency cesarean section [13]. The monitoring of period 2 seems to have inferred an increased adherence to clinical guidelines, measured as a decreased risk of complicated extraction (prolonged duration or more than six pulls or more than one cup detachment). These factors are recurring in some guidelines [1, 3], whilst others stress the lack of evidence regarding concrete limitations [2]. Studies investigating the clinical effect of restricting extractions regarding duration, number of pulls and cup detachment show inconsistent results [6, 26, 27], and the relative importance of avoiding failed extractions vs adhering to guidelines therefore remains an open question.

### Strengths and limitations

The major limitation of the pre-post test study design is arguably the restricted possibility of making causal inference: this design, also known as quasi experimental design, has inherent bias regarding comparison groups due to either a total absence of parallel control group (as in this particular study) or non-randomized control group, as well as differences due to the passage of time. Some confounding is arguably avoided by adjusting for year of birth, but there will still be residual confounding of which we do not know the magnitude. This might include known confounders where we lack data, such as policy changes, staff turnover and proportion of resident vs consultant doctors, as well as variables that we have overlooked. We are also aware of the specific problem of regression towards the mean; normally, an intervention is introduced because of an identified problem, and one can therefore expect a non-representatively extreme value of crucial variables at the study start. With a 19% failure rate among mid and low extractions in period 0, the pre-post comparison is likely to over-estimate the effect size. To overcome this, a prospective study with pre-specified evaluation protocol and a parallel control group would be a suggested alternative method.

In period 1, the imprecision of the components mediating the training effect to the decreased failure rate makes it difficult to interpret the results. Reflecting upon the clinical characteristics of Table 1, the effect was clearly *not* mediated by a population of taller and less dystocic women, nor smaller infants or an increased use of oxytocin. In this material, therefore, we lack an obvious explanatory model. One strength regarding exposure is that the team training set-up contained most of the active components of effective team training identified previously in a review [14].

In exposure period 2, it is reasonable to think that monitoring traction force would make obstetricians more cautious and prone to adhere to guidelines when selecting cases for and performing vacuum extraction. In a monitoring situation, a more cautious selection of candidates for vacuum extraction might be recognized as a decreasing proportion of mid cavity station and non-occiput anterior position extractions from period 1 to period 2, but this is not confirmed by our results. However, the decreased risk of complicated extraction in period 2 might support a possible effect of awareness during monitoring. In a population based study on failed extractions [19], the authors argue that a small increase in failed extractions during a period of educational efforts might actually be an effect of sensible obstetric decisions to consciously convert a difficult extraction instead of employing maximum traction force. Our finding of a partly negative association between the rate of failed extractions and rate of complicated extractions makes a possible illustration of this phenomenon.

## Conclusion

Some of the decrease in failed extractions is likely an effect of active educational effort. The possible monitoring effect of the traction force measurements in period 2 did not seem to deter obstetricians from mid station extractions, but the monitoring may have led to an increased adherence to clinical guidelines.

Despite the limitations to inferring causality in this study design, the results support that alterations in clinical practice should be accompanied by clinical team education.

## Additional files

Additional file 1: Flow chart of included patients. (DOCX 56 kb)

Additional file 2: Description of exposure period 1: team training and vacuum extraction protocol. (DOCX 119 kb)

Additional file 3: **Table S1.** Secondary clinical outcomes. (DOCX 45 kb)
