# Supplementary material for: Effect of team training and monitoring on the rate of failed mid and low cavity vacuum extraction: a hospital based intervention study
Source: BMC Pregnancy Childbirth. 2019 Mar 29;19:101. doi: 10.1186/s12884-019-2257-z (PMC6440163; doi:10.1186/s12884-019-2257-z)
Supplement: Supplementary file 1 — Flow chart of included patients. (DOCX 56 kb) [file 12884_2019_2257_MOESM1_ESM.docx]

Excluded: multiple pregnancy, prematurity <36+0 and double entry in Obstetrics ©

N=49

Included: low and mid vacuum extractions, N= 1074

Eligible: all low and mid vacuum extractions,

N= 1123

Eligible: all vacuum extractions at Karolinska University Hospital during 2007-2008; 2011-2012; 2013-2014, N= 2213

N

Excluded: outlet extractions, N=1090
