# Supplementary material for: Effect of team training and monitoring on the rate of failed mid and low cavity vacuum extraction: a hospital based intervention study
Source: BMC Pregnancy Childbirth. 2019 Mar 29;19:101. doi: 10.1186/s12884-019-2257-z (PMC6440163; doi:10.1186/s12884-019-2257-z)
Supplement: Supplementary file 2 — Description of exposure period 1: team training and vacuum extraction protocol. (DOCX 119 kb) [file 12884_2019_2257_MOESM2_ESM.docx]

The vacuum extraction team intervention programme:

The need to try to decrease the numbers of failed vacuum extractions was discussed at the clinic after a pilot study, showing an incidence of 18 % of failed vacuum extractions among low and mid high VE. None of the outlet VEs failed. An intervention programme was initiated 2009, involving the whole delivery team during VE; physicians (both registrars and specialists), midwifes and assistant nurses, and was carried out annually. The intervention programme included two main items; skills in VE procedure and teamwork. Skills procedure included judgement of fetal station, fetal head position, choice of cup, cup position, create adequate negative pressure, direction of traction and how to protect the perineum. The teamwork included risk factor assessment, team member task assignment, time-out check list, judgement of extraction progress and protection of the perineum. Recommended criteria for vacuum extraction at the department are listed in table 1. The intervention programme is carried out as annually recurring simulated training in vacuum extraction, including one skills training station and one team training station with two realistic vacuum extraction scenarios, and lasted for three hours.

| *Before vacuum extraction* | *During extraction procedure* |
| --- | --- |
| Cervix fully dilated | Rapid negative pressure application up to 80 kPa (one minute) |
| Gestational week 36+0 (34+0) or more | Timekeeping |
| Vertex at level ischial spines or lower | Pull-counting |
| No signs of disproportion | Time limit 20 minutes |
| No fetal coagulopathy or severe maternal blood contamination | Judgement of progress: three pulls to pelvic floor + three pulls from outlet station to cup off |
| Empty bladder | Maximum two detachments of the cup |
| Perineal anaesthesia | Assistant nurse speaks out loud at 3, 6 pulls and 10, 15 and 20 minutes |
| Time-out according to check-list | Kristeller manoeuver not allowed |
|  |  |

**VE-protocol**

Make sure that the negative pressure to 80 kPa is functioning before the suction tube is connected to the vacuum cup!

Pat.ID/Name____________________________________

The obstetrician is in charge of the time out:

1. Presentation of team members incl neonatologist (when present)

2. Adress indication, expected level of difficulty and fetal position/station

3. Empty bladder? PVC? blood samples?

4. Analgesia?

5. Labor enhancement? Obstetrician prescribes level of oxytocin

6. CTG-registration and interpretation. CTG is best seen on pc screen

7. Perineal protection, who is responsible?

Obstetrican/trainee ___________________ Midwife 1 ____________________________

Assistant nurse ____________ Midwife 2 ____________________________

Neonatologist______________________________

Time of cup in place:_____________________+ start timer

Time of negative pressure induction 80 kPa: __________________

(Tip: If time is abundant, induce negative pressure stepwise (20 kPa -> 80 kPa) to check that the cup is free from vaginal mucosa.

Times according to timer:

Pull 1_______________ Pull 2_____________ Pull 3___________NB! State nr of pulls

Pull 4_______________ Pull 5_____________ Pull 6___________NB!

Cup pop off at pull nr :____________________________________________

**NB! State out loud 10, 15 and 20 min respectively from cup application**

CUP REMOVED (time): ___________________________________

FETAL HEAD OUT (time): ___________________________________

DELIVERY (time): ________________________________________

Sign out: summary of procedure, responsibility perineal tear

Documentation is also done in the patient chart Vacuum Extraction template: Time out protocol , timing of any pop-offs, cup position on fetal head, perineal protection – how and by whom?

*Translation of the training documents by Kristina Pettersson and Gunilla Ajne*
