# Supplementary material for: Effect of team training and monitoring on the rate of failed mid and low cavity vacuum extraction: a hospital based intervention study
Source: BMC Pregnancy Childbirth. 2019 Mar 29;19:101. doi: 10.1186/s12884-019-2257-z (PMC6440163; doi:10.1186/s12884-019-2257-z)
Supplement: Supplementary file 3 — Table S1. Secondary clinical outcomes. (DOCX 45 kb) [file 12884_2019_2257_MOESM3_ESM.docx]

| **Table 3. Secondary clinical outcomes**, n(%) | | | | |
| --- | --- | --- | --- | --- |
|  | **Period 0** | **Period 1** | **Period 2** |  |
| **Asphyxia**  **(pH<7.0)** | 6(2) | 4(1) | 16(4) | p^a^ NS  p^b^ NS^**^  p^c^ NS |
| **Apgar <7 at 5 minutes** | 15(5) | 15(4) | 18(5) | NS |
| **Admission to neonatal intensive care unit** | 38(12) | 35(9) | 32(9) | NS |
| **Shoulder dystocia** | 11(3) | 16(4) | 10(3) | NS |
| **Anal sphincter injury** | 41(13) | 51(15) | 61(19) | NS |
| Kruskal-Wallis for multiple testing, pairwise post-hoc test with Bonferroni correction.  ^**^Exact test  a=period 0 vs 1, b= period 1 vs 2, c= period 0 vs 2 | | | | |
